# LabPush: A Pilot Study of Providing Remote Clinics with Laboratory Results via Short Message Service (SMS) in Swaziland, Africa

**DOI:** 10.1371/journal.pone.0044462

**Published:** 2012-09-19

**Authors:** Wen-Shan Jian, Min-Huei Hsu, Hosea Sukati, Shabbir Syed-Abdul, Jeremiah Scholl, Nduduzo Dube, Chun-Kung Hsu, Tai-jung Wu, Vera Lin, Tex Chi, Peter Chang, Yu-Chuan Li

**Affiliations:** 1 School of Health Care Administration, Taipei Medical University, Taipei, Taiwan; 2 College of Medical Science and Technology, Graduate Institute of Biomedical Informatics, Taipei Medical University, Taipei, Taiwan; 3 National Blood Programme and National Clinical Laboratories, Manzini, Swaziland; 4 Institute of Biomedical Informatics, National Yang Ming University, Taipei, Taiwan; 5 Health Informatics Centre, Karolinska Institutet, Stockholm, Sweden; 6 AIDS Healtcare Foundation, Manzini, Swaziland; 7 International Office, Taipei Medical University, Taipei, Taiwan; 8 Taipei Medical University & Hospitals, Taipei,Taiwan; 9 Department of Health, Taipei Hospital, Taipei, Taiwan; Johns Hopkins University, United States of America

## Abstract

**Background:**

Turnaround time (TAT) is an important indicator of laboratory performance. It is often difficult to achieve fast TAT for blood tests conducted at clinics in developing countries. This is because clinics where the patient is treated are often far away from the laboratory, and transporting blood samples and test results between the two locations creates significant delay.

Recent efforts have sought to mitigate this problem by using Short Message Service (SMS) to reduce TAT. Studies reporting the impact of this technique have not been published in scientific literature however. In this paper we present a study of LabPush, a system developed to test whether SMS delivery of HIV related laboratory results to clinics could shorten TAT time significantly.

**Method:**

LapPush was implemented in six clinics of the Kingdom of Swaziland. SMS results were sent out from the laboratory as a supplement to normal transport of paper results. Each clinic was equipped with a mobile phone to receive SMS results. The laboratory that processes the blood tests was equipped with a system for digital input of results, and transmission of results via SMS to the clinics.

**Results:**

Laboratory results were received for 1041 different clinical cases. The total number of SMS records received (1032) was higher than that of paper records (965), indicating a higher loss rate for paper records. A statistical comparison of TAT for SMS and paper reports indicates a statistically significant improvement for SMS. Results were more positive for more rural clinics, and an urban clinic with high workload.

**Conclusion:**

SMS can be used to reduce TAT for blood tests taken at clinics in developing countries. Benefits are likely to be greater at clinics that are further away from laboratories, due to the difficulties this imposes on transport of paper records.

## Introduction

HIV/AIDS is a global health problem. The situation in Africa is particularly severe with HIV/AIDS being the leading cause of death [1]. Blood results are a critical part of diagnoses and management of HIV treatment. CD4 count and percentage for example, are essential parameters in taking care of patients with HIV, and physicians make treatment plans according to laboratory results. Physicians thus appreciate fast and reliable laboratory services [2]. Turnaround time (TAT) is one of the important indicators of laboratory performance [3]. TAT is viewed as an important indicator because it directly reflects on the timeliness of care that can be delivered, and thus on the quality-of-care.

Improvements in laboratory capacity and infrastructure have thus been noted as a general strategy worth exploring for improving treatment of HIV in developing countries [4]. Despite advancement in the use of Information and Communication Technologies (ICT) and transport systems, many laboratories are still struggling to improve their TAT [5]. When health clinics are located at a distance from laboratories one significant issue that can impact TAT is the transport of samples to laboratories and the return of reports to the clinics. The Glasgow Royal Infirmary Laboratory Directorate Audit Committee has reported for example that “slow turnaround times for specimens and reports were not uncommon, but were rarely the result of slow work in the laboratory. They were usually caused by poor arrangements for collecting specimens or returning results. Often these arrangements were not managed by laboratories themselves but they were invariably blamed when things went wrong.”[6]

A related issue is that a number of approaches are being explored to improve HIV treatment with ICT [7]. The rise of mobile communication has also led to the application of Short Message Service (SMS) as a health ICT platform [8]. A high percentage of SMS-based systems for disease prevention in developing countries also focus on support for HIV [9].

Recently this idea has been applied to attempt reducing TAT by sending laboratory results back to clinics using Short Message Service (SMS) [10]–[12]. Reports from projects using this technique have shown positive results for reducing TAT in Kenya and Nigeria [10], [11], and Zambia [12]. Only preliminary reports of their results have been made public however, and no scientifically validated studies (i.e. those published in peer-reviewed journals) have been published based on the results. There thus is still a need for studies that both validate the effectiveness of SMS-based lab reports, and that provide information on how such systems may be improved in the future.

In this study we report results after implementation of the LabPush system in clinics of the Kingdom of Swaziland designed to test whether text-message delivery of laboratory results to mobile phone of the head nurse of the clinics could shorten TAT significantly.

### Background

**Figure 1:**
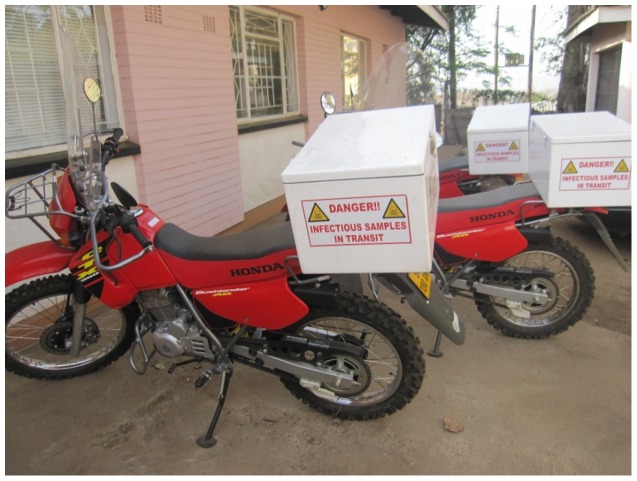
Motor bikes used to transport blood drawn from clinics to the central laboratory.

The situation with respect to reporting and handling of blood tests related to HIV treatment in the Kingdom of Swaziland is similar to the situation described above. The prevalence of HIV in Swaziland is 26.1%, and the population living with HIV was estimated to be 190,000 in 2007 [13]. Patients with HIV receive care through public/private clinics and volunteer counseling and testing (VCTs) facilities in the country. According to the statistics of the National Referral Laboratory (NRL) in Swaziland, there are about 3,000 requests of CD4 tests per month coming from about 40 different clinics. The current system for these tests is that a nurse at the clinic draws the blood of the patient, and this is transported to a centralized laboratory for analysis. Figure 1 shows a photograph of motor bikes used for this purpose. The laboratory then sends a paper record containing the results of the analysis back to the clinic using the same motor bike transport.

Although the central laboratory is able to manage the tests fairly well, and typically can perform the analysis and have the report ready within two days, TAT from when blood is drawn until the report is returned to the clinics is fairly long and unreliable. Usually TAT is approximately 2–3 weeks for many clinics with some exceptions. Because few clinics have TAT only 2–4 days as the number of visits are much more there compared to other clinics.

This paper presents a system called LabPush that has been implemented to reduce the TAT for clinics to receive reports from the lab once they are ready. The system supports sending SMS summaries of lab reports to mobile phones provided to the head nurse at remote clinics. The intent is that this will improve TAT by delivering these reports before motorbike transport can deliver them back to these clinics. This study thus adds knowledge regarding how ICT can be used to improve HIV treatment in developing countries by presenting a more rigorous scientific study of an SMS-based system designed to reduce TAT than has been reported. Although our results show that LabPush can improve TAT and reduce the loss (missing) rate for laboratory reports, further evaluation of the system is still needed.

## Methods

LabPush was developed in the context of the Taiwan Medical Mission (TMM) in the Kingdom of Swaziland that was established in 2008 according to an agreement signed between the two countries. The vision of TMM was to improve medical service and hospital management efficiency in Swaziland by transferring appropriate medical concepts and techniques. Taipei Medical University (TMU) has led the Taiwan Medical Mission in Swaziland since 2009.

In February 2010 TMU sent a group of five medical professionals to the Kingdom of Swaziland. During the visit governmental officers from the Ministry of Health expressed interest in implementing Medical Informatics System (MIS) in order to improve medical services in Swaziland. TMU and TMM thus proposed conducting MIS projects in Swaziland according to the local needs and requirements. The study protocol was approved by the Scientific and Ethics Committee of Swaziland (REF: MH/599C).

### The Design and Development Process

The first phase of developing and implementing these MIS was to conduct an initial investigation in order to assess the most critical needs and requirements of the local healthcare system that could be met through the use of MIS. This was conducted by a research team from TMU that visited different clinics located outside the city of Mbabane to investigate any present problems these clinics face in delivering healthcare. The TMU team consisted of medical doctors and IT staff. The team spent 15 days visiting remote clinics, and talked to various persons including physicians, head nurses and persons in charge of obtaining laboratory results from the laboratory.

During these visits it was revealed that reporting of lab results was a major problem that could possibly be supported through the use of ICT. As noted in the Introduction, the current system for transportation of blood samples and test results utilizes motor bikes between clinics and the central lab. TAT from when tests are requested and when laboratory reports are received at the clinics is increased because sometimes the motorbikes deliver samples and pick up reports from the lab rather infrequently. Normally the clinics store test specimens in a refrigerator until they have a minimum number of blood tests requests, usually 25–30. The samples are then transported to the lab, and the results of the samples that were delivered in the last trip are collected from the lab and returned to the clinic. Every clinic has its own staff for delivering samples and collecting results from the laboratory.

**Figure 2:**
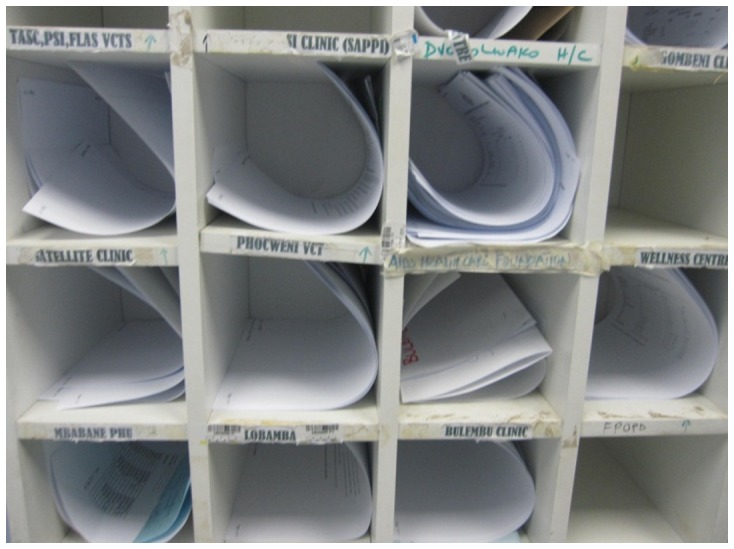
A cupboard where completed lab results are placed while they are waiting to be picked up by staff from clinics.

**Figure 3:**
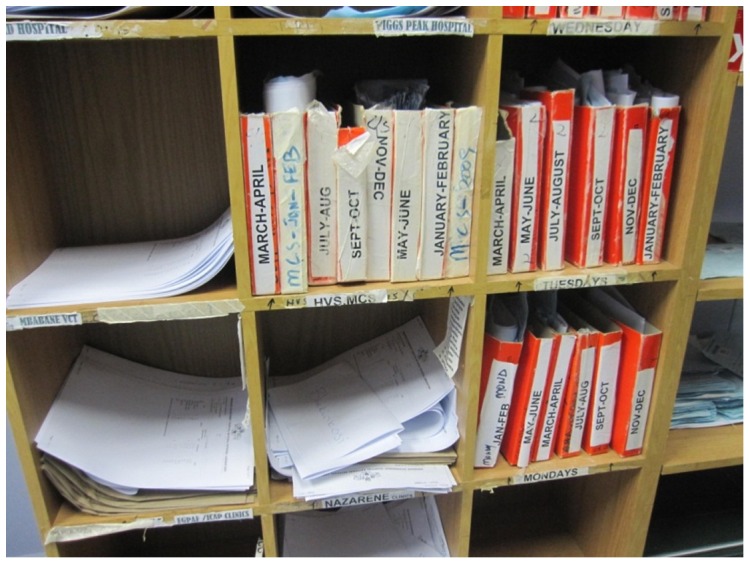
An archive for test results that were not picked up on time by staff members from the remote clinic.

This process can be slow and at times unreliable. Although the lab is able to produce the results within two days after receiving the samples, the time for the results to be delivered back to clinics is quite variable depending on how often the motorbike transport takes trips between the clinic and the lab. This is impacted by factors such as how many samples per day the clinic has been collecting, and how far away the clinic is from the lab. Although official statistics have not been collected regarding the TAT for lab results, doctors working in Swaziland reported to us that peripheral clinics can typically take an additional 2–3 weeks until a motorbike with more blood samples arrives, with other clinics usually fetching the results within 2–3 days. The estimated TAT for each lab is included in Table 1. Figure 2 shows a cupboard at the lab with a pigeon hole for each clinic. Completed lab results are placed in the cupboard while waiting to be picked up by the clinics. In some occasions, it can take quite a while before a clinic picks up the results from the lab pigeon hole, and this can cause the lab to move the results from the cupboard to an archive. Figure 3 shows the archive at the lab that consists of test results that were not picked up on time by one of the clinics.

**Table 1:** The number of paper and SMS records that arrived at least 1 day earlier for each clinic.

Clinic	Distance from lab (km)	Estimated TAT	Paper received	SMS received	Paper arrived first	SMS arrived first	Both on same-day
**1**	25	2–3 weeks	51	51	0	51	0
**2**	32	2–3 weeks	109	109	0	109	0
**3**	22	2–3 weeks	51	51	0	51	0
**4**	18	2–3 days	109	100	68	19	13
**5**	12	2–3 days	199	199	143	30	26
**6**	35	1 week	446	522	76	247	199
**Total**			965	1032	287	507	238
**%**					27.82	49.12	23.06

An additional problem to the long TAT is that for unclear reasons the system is somewhat unreliable and some of the lab results are lost during this process, or during transit. The clinics explained that quite frequently there are occasions where nurses at clinics need to draw blood samples twice due to loss of results during this process. Although they have not collected official statistics as to how often this problem occurs, the medical staff in Swaziland reported to our project members that this situation occurred at a typical clinic perhaps 2–3 times a month. As many of the patients at the clinics have HIV/AIDS and/or TB, the physicians sometimes need to delay treatment while waiting for tests to arrive, and sometimes the test needs to be repeated. The health status of patients can deteriorate during this waiting period.

Based on the information obtained during this visit, and the discussions with the local health staff in Swaziland, a decision was made to go forward with an electronic system. This system, LabPush, would allow for communicating laboratory results electronically to the clinics. Although this would not reduce the amount of time it takes for blood samples to arrive at the laboratory, it would at least have the potential to reduce the amount of time it takes for the clinics to receive the results of the analysis once they are ready.

### System requirements for LabPush

In order to improve the odds for success in the project, one of the authors traveled to Swaziland for 15 days and visited the clinics that would use the system to get design requirements. This is in line with a philosophy used on the project that MIS often need to be customized for each context in which they will be implemented [14], and that this customization needs to be based on deep understanding of the work and care process in the target context [14]–[16]. In order to develop this understanding he conducted interviews and learned about commonly requested lab examinations and possibilities to provide them with lab results as soon as possible once the results are ready in the lab.

Health clinics in Swaziland are not equipped with computers or Internet access which made implementing the system more difficult. However, the clinics do have reliable electricity and are located within range of cellular phone networks. Considering the infrastructure at the clinics, a decision was made to equip the clinics with cellular phones to receive lab results via SMS. The character limitations of SMS did not make it possible to send all the lab information that the clinics required over SMS. After consulting with physicians and nurses at the clinics, ten lab tests were chosen that are essential for the physicians to take clinical decisions, and make treatment plans, before the paper results arrived. The 10 tests selected to send via SMS are; aspartate aminotransferase (AST), alanine aminotranferease (ALT), indirect bilirubin (I-bil), total bilirubin (t-bil), serum creatinine (Cre), heamoglobin (Hb), platelets (Plt), white blood corpuscles (WBC), absolute CD4 count (CD4), the percentage of CD4 count (CD%) and the percentage of lympocytes (LY%). Because SMS can only use 140 characters the abbreviations noted above for each of the blood tests were used to cut the number of the SMS characters. Because paper records are still an integral part of the overall information infrastructure at the clinics and laboratories, a decision was made that paper records would still be sent from the labs to the clinics while the LabPush system is in use. Therefore the LabPush system was specified to supplement rather than replace paper records.

A mobile phone with a SIM card would be provided to each participating clinic, and the use of this mobile phone would be restricted to only receiving lab results via SMS. The message language was decided to be English, the official language of Swaziland. The SMS content also included the patient name, bar-code number sent when the test was ordered, and the results of the tests.

After the visits to the clinics and discussions with health staff, the author involved in the information and requirements gathering returned to Taiwan and provided input to a software development team at TMU. During the software development phase not very much communication and participation was elicited from the clinical staff in Swaziland. Although it is generally recommended to use participatory design processes that allow interactive prototyping for MIS [17], in this case it was not deemed practical. The risks were also lower than for most MIS projects since the system was fairly uncomplicated both technically and in terms of the interaction that needed to be supported, and because the design leader that visited Swaziland was a medical doctor with experience working in developing countries. His clinical education and experience, and previous field work on site, made it highly likely that he would be able to oversee the development process without needing to obtain interactive design information from the users.

### Implementation of the system

After the software development process was finished, the system was implemented at the lab and six clinics. Three of these clinics were located within 50 km of the lab and three of them were located 50–100 km away. The system implemented at the laboratory consisted of a miniserver and MySQL software. The results usually became available on the Laboratory Information system (LIS) within 2–3 days after a sample was delivered to the lab. The LIS was a preexisting system that was not created as part of the LabPush project. One of the staff from laboratory was assigned to extract the results from LIS into an Excel file and uploaded it into the LabPush system. Software was developed as a part of LabPush that can extract the clinic ID, patients ID, age, and test results from the Excel file. This information was automatically translated into an SMS and sent to the cellular phone number of that clinic. The end-users are head of clinics that use the labPush system. And this system does not need any special training just to read SMS and write in log book. However, half day training was provided to the staff from lab about the procedure of manually extracting excel file from LIS and uploading in to the LabPush system.

**Figure 4:**
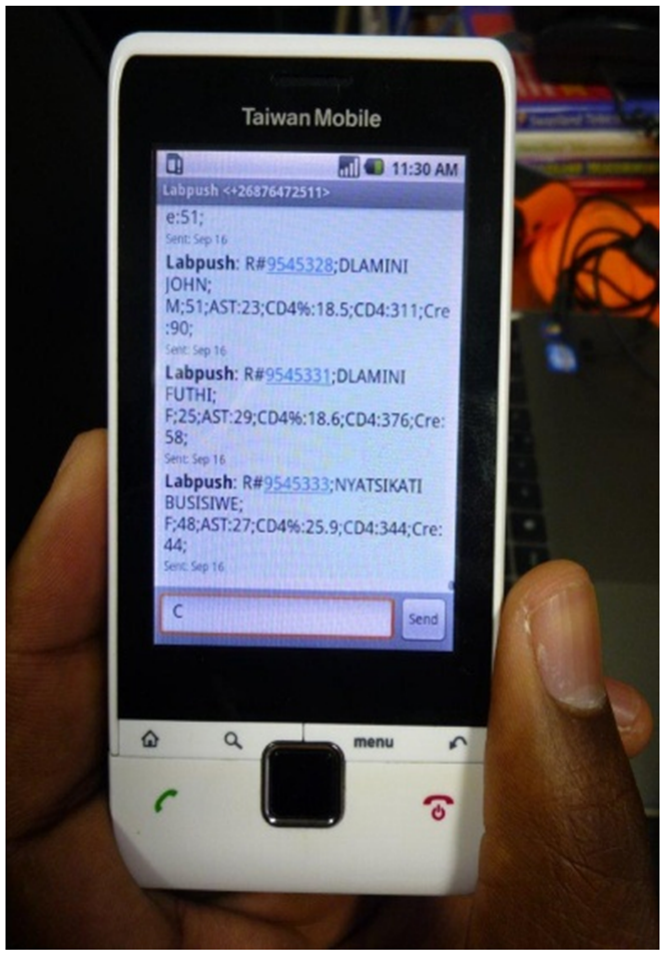
Diplay of mobile phone with received laboratory results via SMS.

The workflow that is required to support the process above is not insignificant. In order to reduce the impact on workflow required for sending SMS reports from the laboratory, it was thus decided that records would not be sent out immediately once they are available. Instead the workers at the lab wait until enough of them have accumulated, and then send them out in bulk. Generally this occurs every 8 days. The number of days to send SMS reports can be adjusted according to the local requirement. In this study we decided to send SMS reports oncea week. Figure 4 shows a snap shot of a phone that receive laboratory results via SMS as part of the LabPush system. Six out of fourteen clinics that expressed interest to participate in the project were selected randomly for the first pilot phase.

### Data collection

The head nurse at each participating clinic was requested to maintain a log book to record the laboratory results and the time they received the SMS and paper results. Written consent was obtained from the head nurse of each participating clinic in order to obtain data related to the study. The head nurse at each of the participating clinics compiled the date when lab results arrived through SMS, or paper results from the laboratories arrived to the clinic. Data presented in this study was collected for three months from September to November 2011.

## Results

During the study period laboratory results were received for a total of 1041 different clinical cases. Table 1 shows the total number of laboratory results received for both SMS and paper records at each clinic. The number of records for each type that arrived at least one day before the other type of record is also included. The data indicates that SMS records arrived before paper records 49.12% of the time, and that paper records arrived first 27.82% of the time. In addition the total number of SMS records received (1032) was higher than that of paper records (965), indicating a higher loss rate for paper records. These numbers indicate a generally better performance for SMS records than paper records.

Table 2 shows the number of days gained for each type of delivery in cases where one delivery method arrived before the other. These results show that the four clinics further away from the laboratory did have quite a large improvement with SMS delivery, since the minimum number of days that were saved with SMS was 21 days. Thus, it took at least three weeks for the paper record to arrive once the SMS record had arrived, and the mean savings being at least 23 days at each of these clinics. However, the difference between SMS and paper records was much less at the clinics closer to the laboratory. Although paper records often performed better than SMS, the delivery of SMS every 8^th^ day meant that the maximum savings for paper records over SMS could be 8 days only.

**Table 2:** The average gain in number of days for each delivery method for cases where one record arrived before the other.

	Clinic	N	Minimum gain	Maximum gain
**Paper**	1	51	0	0
	2	109	0	0
	3	51	0	0
	4	109	0	4
	5	199	0	8
	6	446	0	2
	Total	**965**	0	8
**SMS**	1	51	21	29
	2	109	21	29
	3	51	21	26
	4	100	0	3
	5	199	0	4
	6	522	0	10
	Total	**1032**	0	29

## Discussion

The results of this study indicate that SMS based reporting of lab results has promise for reducing the TAT, and loss (missing) rate for sending lab reports to clinics in settings that use physical transport of paper records. When comparing SMS delivery to paper delivery, both the total number of results received by the clinics and the number received first (i.e. that arrived faster) was significantly better for SMS than the paper based system. SMS based systems thus have the potential to allow patients to begin treatment, and have their treatment adjusted more rapidly than with the use of paper reports alone. An additional finding of the study is that the loss rate for paper records during our study was at least 3.7% (based on the fewer number of paper records that arrived than SMS records). This is possibly a little bit higher than that, since there might have been some cases in which both the paper record and SMS were both lost. The estimates by the medical staff of lost reports occurring only 2–3 times a month at a typical clinic indicates the actual number of lost reports is probably not much higher than we reported however. The general loss rate is possibly a bit higher than current informal estimates, since we noticed an average of over 10 paper records lost per clinic during our month long study. Since official statistics have not been reported regarding the loss rate of reports, this can serve as a starting point on motivating future research on how to improve the handling of paper records from the labs to the clinics.

When looking at the situation for each of the respective clinics, the performance is somewhat mixed (see Table 1). The two clinics that were closer to the laboratory did have a better performance for paper records than SMS records. This can be explained because, being close to the laboratory, these clinics have an easier time fetching paper records, and thus the SMS delivery on every 8^th^ day was not frequent enough to provide generally better performance than their usual paper record delivery. However, the number of days to send SMS reports can be adjusted according to the local requirement. This indicates that SMS based lab report systems should be implemented with caution if they are going to be a replacement for paper records at clinics that are fairly close to labs.

The benefits for the four clinics located further away from the laboratory were quite significant during the study, with SMS allowing the information to reach the clinics over three weeks before paper records could be delivered in many cases. Since the study did not include an estimate of costs, a cost benefit analysis of the system for each of these contexts will still need to be conducted in order to identify the overall benefits that can be expected from the system.

There are no major threats to the security and confidentiality of the lab results transferring via SMS, since SMS is encrypted and secure. The only possibility to steal the patient information would be from the lab or at the clinic after it has arrived. Thus, this does not increase the security risk in comparison to paper records. SMS is sent only to the mobile phone provided to the head nurse of each clinic. The head nurses are instructed to copy the SMS results in the log book and delete the message. Even in case, if a mobile phone is stolen from the clinic the nurse can alert laboratory to stop sending SMS to that mobile phone. Thus, the patients' lab results can be prevented from breaching.

### Further development of the LabPush system

Since this study reports a preliminary investigation, and MIS should generally be improved in an iterative fashion based on information obtained from preliminary implementations of the system [18], it is worth discussing how the information obtained from the study will help inform the future direction of the LabPush system in Swaziland, and can aid the designers of similar systems in other contexts. Although the system in its current form was not designed to be a replacement for paper records, the benefits at the remote clinics were significant enough to make it interesting to consider if further development of the system to suit this purpose would be worthwhile. Using SMS as a supplement to the paper record system will generally incur extra costs on the treatment process, and thus any benefits that are gained will need to be considered in the context of other options that may be available to improve care. If SMS can be used to replace paper record transport however, then the cost savings related to sending motorbikes etc. can also be factored into these calculations, and the potential for improved service at lower cost for SMS compared to paper may be possible.

One of the challenges that such a redesign would present however, is that the clinics currently do not use electronic records in their day-to-day work process. Replacing paper transport of records with SMS transport would thus require either that the workflow at the clinics be entirely redesigned to support work with electronic records, or for printing mechanisms to be developed and implemented so that the SMS records could be printed out and used for the same purpose as the current records transported via motorbike. Other studies have investigated the use of SMS printers for this purpose.

An additional issue is that the system will need to be adapted so that more complete information could be obtained from the SMS based delivery, instead of just a summary of a few critical values. The better performance for paper records compared to SMS at clinics closer to the lab also indicates that, for these clinics a more beneficial approach would be to focus on improving the workflow at the laboratory so that it is possible to send out SMS reports more quickly. Without workflow improvement caution should be taken when trying to replace paper record transport with SMS, as TAT may actually end up worse for these clinics. In addition to generally making it possible for clinics close to the laboratory to see better results for SMS reports than paper reports, these improvements should also make it possible to improve the TAT even further for clinics further away from the laboratory. Efforts to improve the workflow at the laboratory are also is consistent with suggestions that efforts to reduce TAT should look at all the different issues that impact TAT, and iteratively improve them over time [19].

Efforts in the future can also be made to consider how to make SMS delivery system more reliable, for example by validating if reports have been received, and by resending the SMS when they have not. When considering how to generalize this service for other contexts, one issue worth considering is that SMS is fairly expensive, and cellular phone networks need to be available at all clinics that will use it. In the case of this project we are able to purchase SMS services in bulk in order to reduce costs, but in some situations the costs of SMS bandwidth could be a significant portion of the overall service costs. In many rural areas of Africa affordable cellular phone networks will also not be available in the near future [20]. Thus it would be interesting to explore a system that uses point-to-point WiFi [21], [22], or Delay Tolerant Networking (DTN) [23], [24] as a substitute for SMS in situations where cellular phone networks are not available for usage by the healthcare system, or to provide the service at lower cost than SMS. Additionally, these mechanisms could be used to transport much larger records so that all relevant information, including medical images, could be included in the report. The development of inexpensive DTN-based printers similar to the SMS-printer used in similar studies should make this option viable.

### Limitations

The methodology utilized for this study has several limitations because it was designed to give an initial indication of the possibility for SMS to improve the laboratory reporting process, while also reducing the amount of time used by health workers to collect data. For these reasons the day that blood tests were sent from the clinics, and also that tests were made available at the laboratories were not recorded. It is thus not possible for example, to determine the loss rate of SMS and paper records in situations where both the paper and SMS record were lost. It is also not possible to identify the overall reduction in turnaround time from the day that the reports are available at the laboratory until the day that the reports arrive at the clinics. Although we did incorporate some very rough estimates of these issues into our analysis, this estimated data cannot be considered highly reliable. Future studies will focus on these issues in order to provide more accurate figures.

An additional consideration is that this study did not look into the overall cost-benefit analysis for the system when considering issues such as the hardware and software costs for the service, and reductions or savings in workflow, in comparison to any improvements in treatment. More comprehensive studies that consider all of these issues need to be conducted in the future before clear recommendations can be given on overall benefits.

### Conclusions

This study provides preliminary evidence that SMS reporting of critical lab results to clinics has improved TAT of the national laboratory in Swaziland. Missing rates of test results were reduced significantly. Benefits from this system were noted to be greater at clinics that are further away from the lab. Further studies are needed in order to learn how to improve services of this type, and to provide more clear evidence of clinical benefits while also considering the costs of the service. Efforts to improve the workflow at labs so that SMS reports can be sent out more often, and to develop DTN-based systems that can be used when SMS is not available, are also interesting avenues for future research. Caution should be taken when trying to replace paper records with SMS records at clinics close to labs if the workflow at the lab for sending paper records has not been optimized, as in that situation it is possible that the SMS reports will result in worse TAT than paper reports.
